# The complete mitochondrial genome of giant cricket, *Tarbinskiellus portentosus* (Orthoptera: Gryllidae) and its curation

**DOI:** 10.1080/23802359.2022.2107441

**Published:** 2022-08-05

**Authors:** Somjit Homchan, Yash Munnalal Gupta

**Affiliations:** Department of Biology, Faculty of Science, Naresuan University, Phitsanulok, Thailand

**Keywords:** DNA barcoding, Edible crickets, Food security, Giant cricket, Mitochondrial genome

## Abstract

*Tarbinskiellus portentosus*, commonly known as giant cricket one of the important edible cricket species. However, the genetic information of these species is still limited. Therefore, we have assembled and annotated the first mitochondrial genome of *T. portentosus*. The mitogenome is 15710 bp long and has GC content of 27.19%. The nucleotide composition is similar with other insect mitogenomes (A 40.6%; T 32.2%; C 17.3%; G 9.9%). The gene organization in the mitogenome of *T. portentosus* is identical to the mitogenome of other cricket species. The complete mitogenome of *T. portentosus* consisted 37 genes including 13 protein coding genes, 22 tRNA genes, and two rRNA genes. The newly assembled mitogenome will help molecular biology research on edible crickets. Since mitogenome genes are traditionally used for DNA barcoding and phylogenetic analysis, comparative analysis of

*T. portentosus* mitogenome with other related cricket species will also aid researchers in developing universal primers for species identification toward food security. Apart from the main goal of providing full mitogenome of *T. portentosus,* paper also provides conceptual workflow based on *de novo* assembly and its correction for final mitogenome construction.

## Introduction

*T. portentosus* also known as *Brachytrupes portentosus* has become an important edible cricket species in Thailand (Gupta et al., 2020). These crickets are large; therefore, they are usually referred to as giant cricket. These species are difficult to raise in farms because crickets burrow holes in land fields and reside beneath the surface (Nischalke et al., 2020). Thai locals collect these crickets by identifying them morphologically (Hanboonsong, 2010). However, it cannot be the optimal approach for industrial scale production and food security. Additionally, the entomophagy is increasing due to high nutritional value of edible crickets, but molecular research is still limited considering the popularity of edible insects (Van Huis, 2020). Even, the until present study, only partial *cox1* sequences of *T. portentosus* was accessible. Consequently, the first complete mitogenome of *T. portentosus* is assembled and annotated using reproducible bioinformatic pipeline.

The tool and scripts used in bioinformatic workflow presented in study have been utilized for mitogenome assembly and annotation. However, the final assembled sequences often needs error correction to get rid of sequence artifacts and ambiguous nucleotides (Baker, 2012). Therefore, we have proposed the realignment of mitogenome assembly against raw sequence reads. Repeats in the genome are an error-prone region that makes sequence assembly challenging (Parra et al., 2009). Errors in sequence reads sometimes becomes nearly impossible to detect or remains unclear even after correcting them with k-mer based algorithms (Kelley et al., 2010). A recent article examined inaccuracies in mitochondrial genome data provided to NCBI (Prada & Boore, 2019). The research community may be misled by incorrect genome sequence assembly and annotation. In case of assembling small mitochondrial genomes using *de novo* assemblers, the final assemblies can be inspected by realigning them with sequence reads. Therefore, this study also seeks to authenticate the successful mitochondrial sequence assembly by realigning them with their raw reads.

The assemble mitogenome will also be a reference for assembling new mitogenomes for other related cricket species using mitochondrial genome assemblers like NOVOplasty (Dierckxsens et al., 2017). Methodology presented in current study will serve an alternative approach for researchers to assemble and curate the mitogenomes.

Moreover, the newly assembled mitogenome will contribute to genetic research on edible crickets for enhancing food security in mass rearing facilities. To be more precise, the mitogenomic regions will serve as a temple for *T. portentosus* identification and other related cricket species.

## Materials and methods

### Sequence assembly

The paired-end fastq genomic sequences were obtained from sequence read archive

(Organism: *T. portentosus*; BioSample: SAMN19844586; SRA accession number: SRR14902953; Submitter: Research Institute of Resource Insects; Geographic location: China, Guangxi, Baise). The forward and reverse reads quality for mitochondrion assembly was checked by FastQC (Brown et al., 2017). The total spots reads were 18,308,560. Both reads were trimmed using Trimmomatic tool (Bolger et al., 2014) to elevate the increasing eliminate bases from a read if it falls below a quality level and to remove the adapter sequences. The trimmed sequences were 18, 057, 997 for forward and reverse reads. Read quality was re-checked prior to assembly. Trimmed forward and reverse reads were used for *de novo* assembly using NOVOplasty perl script (Dierckxsens et al., 2017). The known partial sequence of *T. portentosus* cytochrome c oxidase subunit I (*cox1*) gene (Accession number: MT429708) was used for initiating the assembly.

### Sequence assembly correction

The single circular assembled sequence generated by the NOVOplasty perl script (Dierckxsens et al., 2017) was examined for the presence of ambiguous nucleotides (N) and sequence assembly errors. To replace the ambiguous nucleotide with the correct nucleotide, the section of sequence containing the problematic nucleotide was cut and re-aligned to the raw reads. Therefore, the nucleotide sequence section containing ambiguous nucleotides from circular assembled sequence was aligned to raw reads (fastq) from sequence read archive (SRA accession number: SRR14902953) using basic local alignment search tool (BLAST)(Altschul et al., 1990).

### Genome annotation and visualization

The final sequence assembly was annotated using MITOS (Bernt et al., 2013). The assembled mitochondrion was aligned other nucleotide sequences BLAST (Altschul et al., 1990) for secondary authentication of annotated CDS features. tRNAscan-SE (Chan & Lowe, 2019) was used to confirmed the tRNA annotations found in the mitochondrion. 22 tRNA genes were also inspected manually to check the present of anticodons for respective amino acids. Annotated mitochondrion of *T. portentosus* was submitted to NCBI and nucleotide sequence data reported are available in the Third Party Annotation Section of the DDBJ/ENA/GenBank databases under the accession number TPA: BK059220. The GenBank file generated and given by NCBI was used to visualize mitochondrial genome map by OrganellarGenomeDRAW (OGDRAW) version 1.3.1 server (Greiner et al., 2019).

### Phylogenetic analysis

A total of 13 mitogenomes of related cricket species and one previously published

*T. portentosus* mitogenome (MZ427921.1) and one *Tarbinskiellus* spp. mitogenome were aligned with the *T. portentosus* mitogenome assembled in the present study (Accession number: BK059220) using MUSCLE (Edgar, 2004). The substitution pattern was best described by models with the lowest BIC scores (Bayesian Information Criterion). Therefore, the best fit model was estimated by jModelTest Version 2.1.10 (Darriba et al., 2012) using BIC. The general time reversible model with gamma distributed with invariant sites (Nei & Kumar, 2000) (GTR + G + I) model was used for analysis because of lowest BIC score compared to other 88 different nucleotide substitution models. Phylogenetic tree was contrasted using Bayesian inference (BI) with BEAST Version 2.6.6 (Drummond et al., 2012) with Markov chain Monte Carlo (MCMC) 100 million generations and visualized by FigTree Version 1.4.4 (Rambaut, 2009).

## Results and discussion

An initial objective of the present research was to assemble whole mitochondrial genome of *T. portentosus* to provide longer gene regions for DNA barcoding purposes. Therefore, the methodology of this study was designed to employ the raw sequence data for the assembly. In total, 109,292 reads were used in the final assembly, resulting in a circularized sequence of 15,710 bp with a GC content of 27.19%. The average coverage of the mitochondrial DNA was 1253x. Only three ambiguous nucleotides were detected in the final assembly. Therefore, the section of mitochondrial assembly containing these ambiguous nucleotides was realigned against raw sequence data. The correct nucleotides were added manually depending on their coverage for respective base position in the mitochondrial sequence. Conducting *de novo* assembly can be complicated due to presence of repeated nucleotides in the sequence. Therefore, the final assemble sequence should be inspected before the annotation or downstream process. The inspection should follow the steps of realigning assembled sequence to raw reads. Realignment can be performed on local machine or using BLAST (Altschul et al., 1990) on NCBI if dataset is already deposited to sequence read archive (SRA). Herein, we have used online method to fasten the realignment process using SRA dataset (Accession number: SRR14902953). The ambiguous nucleotide containing region of assembled sequence was realigned to read from SRA to resolve the unclarity of three ambiguous nucleotide which were located around single repeats. Moreover, we found long strands of repeated sequences before those single repeats. Therefore, regions containing repeated sequence with unique flanking sequences were realigned with reads to confirm the final assembly. We strongly advise that, even if the respected *de novo* assembler (e.g., NOVOplasty) (Dierckxsens et al., 2017) generates the appropriate assembled sequence, the sequence should be scrutinized, particularly in regions containing repeats. For instance, the available *T. portentosus* mitogenome (Accession number: MZ427921) on GenBank has only 20x coverage and also lacks the repeated region from D loop, making it smaller than the mitogenome assembled and curated in the current study. Herein, we demonstrated the way of curate the erroneous assemblies generated due to sequence repeats by comparing it with raw sequence reads. Majority of repeated sequences present in control region of insect mitochondrial DNA (Ji et al., 2019; Zhang & Hewitt, 1997) as we also observed in present mitochondrial genome of *T. portentosus*.

**Figure 1.**
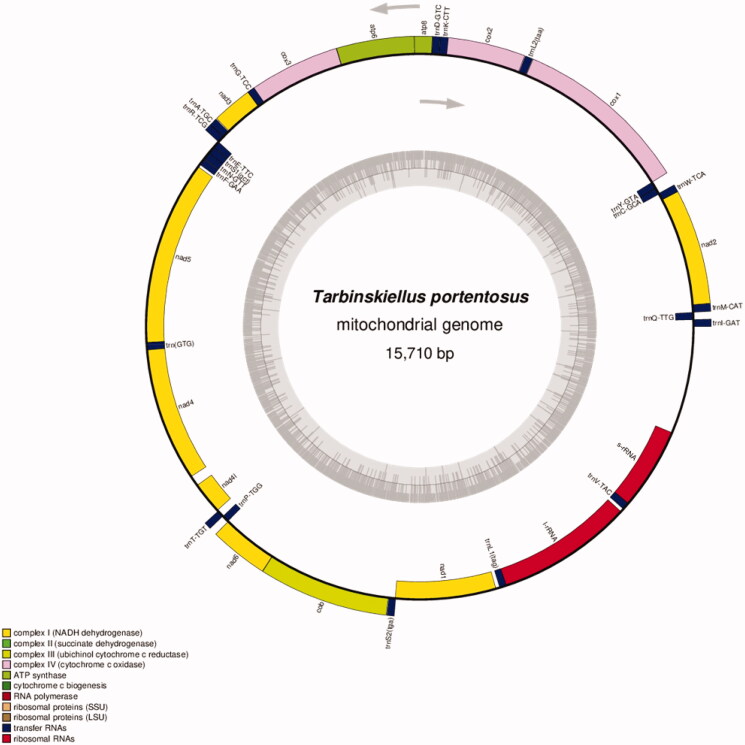
The complete mitochondrial genome map of *T. portentosus.* Arrangement of 37 genes represented in the map, including 13 protein coding genes, 22 tRNA genes, and two rRNA genes. The GC% along the mitochondrion is represented by the inner circle.

The final assembled sequence was having 37 genes. The respective order of these genes (13 protein coding genes, 22 tRNAs, 2 rRNAs): Ile, Gln, Met, *nad2,* Trp, Cys, Tyr, *cox1,* Leu-2*, cox2,* Lys, Asp, *atp8, atp6, cox3,* Gly, *nad3,* Ala, Arg, Glu, Ser-1, Asn, Phe, *nad5, nad4,* His, *nad4l,* Thr, Pro, *nad6, cob,* Ser-2, *nad1,* Leu-1, L-rRNA Val, S-rRNA. The gene order and direction were comparable with mitogenomes of related cricket species from same subfamily of Gryllinae (*Loxoblemmus doenitzi:* NC_033985.1*, Teleogryllus emma:* NC_011823.1). Annotated mitochondrial genome map of *T. portentosus* is shown in Figure 1.

**Figure 2.**
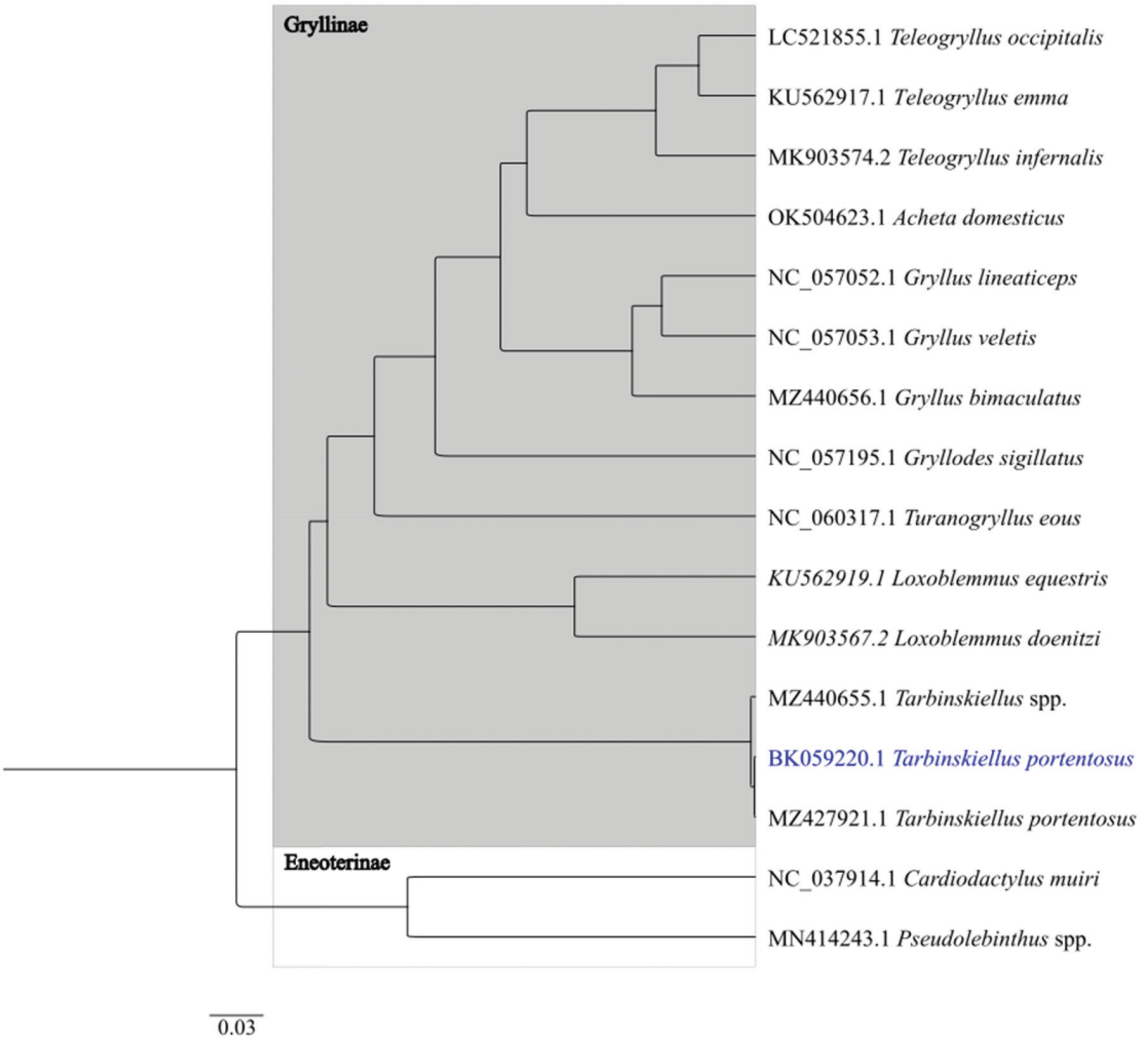
The evolutionary history was inferred using Bayesian inference and General Time Reversible model. The analysis involved 14 mitochondrion sequences. *Cardiodactylus muiri* and *Pseudolebinthus* species were taken as outgroups.

The phylogenetic analysis was conducted in over to position *T. portentosus* with other related species of crickets using whole mitochondrial DNA (Figure 2). *T. portentosus* grouped within the monophyletic subfamily of Gryllinae. Other two cricket species belong to subfamily of Eneopterinae, which out-grouped and clustered in single clade. Result suggested that *T. portentosus* has close relative with *Loxoblemmus* and clustered in same clade. The presented phylogenetic analysis is clearly consistent with earlier findings and strongly supported sub-familial monophyly (Ma et al., 2019).

Recently published study used 16S rRNA gene for genetic variation study of

*T. portentosus* from different location in Thailand. In their study, researchers found it difficult to amplify the *cox1* sequence from *T. portentosus* DNA samples for their investigation (Pradit et al., 2021). In future, researchers will be able to use the entire mitochondrial sequence given in this paper to undertake genetic investigations on *T. portentosus* and other edible crickets. On other hand, DNA barcodes have been developed from mitochondrial DNA for edible crickets (Gupta et al., 2020). Although entomology is becoming increasingly prominent, genetic information on edible insects for DNA barcoding and population genetics is still limited. Therefore, the mitochondrial DNA sequence information presented will aid in DNA barcoding and confine phylogenic position of *T. portentosus.* On the other hand, by examining the reproducibility of the provided methodology, it may provide a faster approach for mitochondrial genome assembly and error correction in contigs produced by DNA sequence assemblers.

## Data Availability

The assembled mitochondrial genome sequence which supports this study is available at NCBI (https://www.ncbi.nlm.nih.gov/). Nucleotide sequence data reported are available in the Third Party Annotation Section of the DDBJ/ENA/GenBank databases under the accession number TPA: BK059220.
